# Kinetic characterization of human mRNA guanine-N7 methyltransferase

**DOI:** 10.1038/s41598-024-55184-5

**Published:** 2024-02-24

**Authors:** Sumera Perveen, Aliakbar Khalili Yazdi, Taraneh Hajian, Fengling Li, Masoud Vedadi

**Affiliations:** 1 Structural Genomics Consortium, University of Toronto, Toronto, ON M5G 1L7 Canada; 2 Ontario Institute for Cancer Research, 661 University Ave, Toronto, ON M5G 0A3 Canada; 3 Department of Pharmacology and Toxicology, University of Toronto, Toronto, ON M5S 1A8 Canada; 4 QBI COVID-19 Research Group (QCRG), San Francisco, CA 94158 USA

**Keywords:** Cancer, Drug discovery, Biochemistry, Enzymes, RNA

## Abstract

The 5′-mRNA-cap formation is a conserved process in protection of mRNA in eukaryotic cells, resulting in mRNA stability and efficient translation. In humans, two methyltransferases, RNA cap guanine-N7 methyltransferase (hRNMT) and cap-specific nucleoside-2′-O-methyltransferase 1 (hCMTr1) methylate the mRNA resulting in cap0 (N7mGpppN-RNA) and cap1 (N7mGpppN2′-Om-RNA) formation, respectively. Coronaviruses mimic this process by capping their RNA to evade human immune systems. The coronaviral nonstructural proteins, nsp14 and nsp10-nsp16, catalyze the same reactions as hRNMT and hCMTr1, respectively. These two viral enzymes are important targets for development of inhibitor-based antiviral therapeutics. However, assessing the selectivity of such inhibitors against human corresponding proteins is crucial. Human RNMTs have been implicated in proliferation of cancer cells and are also potential targets for development of anticancer therapeutics. Here, we report the development and optimization of a radiometric assay for hRNMT, full kinetic characterization of its activity, and optimization of the assay for high-throughput screening with a Z-factor of 0.79. This enables selectivity determination for a large number of hits from various screening of coronaviral methyltransferases, and also screening hRNMT for discovery of inhibitors and chemical probes that potentially could be used to further investigate the roles RNMTs play in cancers.

## Introduction

The newly formed RNA transcripts undergo a methylation process called capping, which was first described in 1970s^1^. The capping process is conserved among various organisms and protects the mRNA from immune systems, and increases their stability during translation^2^. RNA capping is essential for eukaryotic cell growth and is a multi-step enzymatic process^3^. First, an RNA triphosphatase removes an inorganic phosphate from the 5′-triphosphate terminus of the newly transcribed RNA. Then, an RNA guanylyltransferase forms a guanylate cap^4^. The next step involves the methylation of guanine at N7 position by an RNA cap guanine-N7 methyltransferase (GN7-MTase)^5,6^. GN7-MTases are conserved among eukaryotes and most of infecting viruses, and are necessary for viral survival. In humans, the RNA guanine-N7 methyltransferase (hRNMT) is the GN7-MTase responsible for creation of 5′-N7mG-mRNA.

Human RNMT is encoded by the *RNMT* gene with a subcellular location in the nucleus and Nucleoli fibrillar center. It is a 476 amino acid-containing protein with a molecular weight of 55 kDa^7^. The N-terminal domain (1–120 amino acids) of human RNMT is not required for catalytic activity, rather it regulates RNMT activity by facilitating the recruitment of RNMT to RNA polymerase II transcription initiation sites^8^. The catalytic domain of RNMT lies between residues 121–476, and is conserved in sequence, structure, and function in all mRNA cap methyltransferases^4^. RNMT contains three nuclear localization signals (NLS), two of which are located on N-terminal domain while third is within its catalytic domain^9^. A small RNMT activating mini protein (RAM) acts as an activating subunit for RNMT at the transcription site. RAM is required for recruiting RNMT at the transcription site and is essential for cell survival but does not affect the methyltransferase activity of RNMT, helping in transcription and RNA stability^10^. Another interacting partner for RNMT is the eukaryotic translation initiation factor (eIF4E), which is one of the major N7Gm cap-binding proteins in mammalian cells. eIF4E binds to RNMT to regulate the capping process^11^.

Interestingly, the mRNA translation is dysregulated in many cancers, this raises the potential of utilizing the mRNA capping enzymes as therapeutic targets to selectively inhibit protein synthesis in cancer cells^12^, where RNMT playing a major role. RNMT is a promising therapeutic target in PIK3CA (phosphatidylinositol-4,5- bisphosphate 3-kinase catalytic subunit alpha gene) mutant breast cancer, as PIK3CA mutations in breast cells depend on RNMT (mRNA cap methylation) for their survival and proliferation^13^. Reduction of cellular activity of RNMT increases apoptosis of breast cells by reducing their proliferation without affecting the proliferation of non-transformed mammary epithelial cells^13^. In malignant brain tumors (Glioma), B7-H6 (B7 homologue 6) is expressed abnormally and RNMT expression was significantly decreased in B7-H6 knock-down glioma stem-like cells (GSLCs) suggesting RNMT role in B7-H6 tumor cell proliferation enhancement via the c-Myc/RNMT Axis. Therefore, B7-H6/RNMT could be targeted for glioma therapy^14^. Taken together, RNMT plays a critical role in tumorigenesis and it is a valid target for development of cancer therapeutics.

On the other hand, GN7-MTases are also attractive targets for antiviral and antifungal drug development^15–18^. For example, numerous viral genomes, such as coronaviruses, encode N7-MTases responsible for viral RNA capping. These enzymes enable the viruses to evade the immune systems in humans, and therefore, they have been considered valuable targets in the discovery of antiviral therapeutics^19–23^. Although viral and human RNA cap structures are quite similar, still the viral RNA capping enzymes show some differences from host cells in respect to organization of their genes, subunit composition, and structure^24^. Hence, the pathogenic cap‐forming enzymes are potential targets for antiviral drug developments^20–22^. However, in these cases, the availability of selectivity assays against human GN7-MTase is critical for develop of potent and selective antiviral drugs.

Here we report the development and optimization of a radiometric assay for human RNMT, its kinetic characterization, and optimization for high throughput screening. This assay is suitable as a primary assay for screening RNMT to identify inhibitors towards development of anticancer drugs, and performing selectivity assays for inhibitors identified against pathogenic N7-MTases, such as SARS-CoV-2 nsp14.

## Results and discussion

### Assay development and optimization

**Figure 1:**
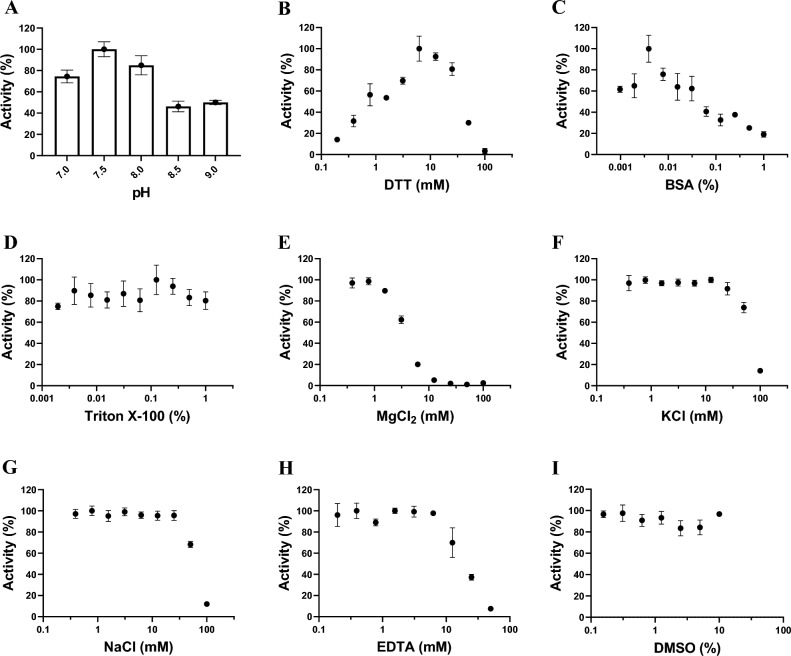
Assay optimization for RNMT. (**A**) RNMT activity was tested at a pH range of 7.0 to 9.0 using 10 mM Tris–HCl. The effects of additives such as (**B**) DTT, (**C**) BSA, (**D**) Triton X-100, (**E**) MgCl_2_, (**F**) KCl, (**G**) NaCl, (**H**) EDTA, and (**I**) DMSO were evaluated using optimal pH buffer (10 mM Tris, pH 7.5). Plotted values are the mean ± standard deviation of three independent experiments. Data were analyzed using GraphPad Prism 9.

Human RNMT (1–476) was purified (Suppl. Fig. S1) and its activity was tested in vitro by monitoring the transfer of ^3^H-SAM to the biotinylated RNA substrate. An initial experiment with 5 nM RNMT concentration, 150 nM RNA substrate, and 250 nM ^3^H-SAM indicated that RNMT is active and reaction components have a good signal-to-noise ratio to be used for further optimization. The assay conditions were further optimized with respect to pH of the buffer and the presence of several commonly used additives (Fig. 1). The highest RNMT activity was observed in Tris–HCl buffer at pH 7.5 (Fig. 1A). The effects of other buffer components like DTT, BSA, Triton X-100 were investigated at pH 7.5. An increase in activity was observed with up to 10 mM DTT (Fig. 1B). The presence of BSA at concentrations higher than 0.01% significantly reduced the enzyme activity (Fig. 1C), while Triton X-100 had no effect on RNMT activity up to 1% (Fig. 1D). The presence of MgCl_2_ at concentrations above 1.5 mM led to a significant decrease in activity (Fig. 1E). KCl also reduced RNMT activity at 25 mM by about 30% (Fig. 1F). A similar effect was observed for NaCl (Fig. 1G). EDTA also reduced the activity of RNMT at concentrations above 10 mM (Fig. 1H). Based on these optimization results, final assay conditions selected for RNMT activity assays and kinetic characterization were 10 mM Tris–HCl pH 7.5, 250 µM MgCl_2_, 10 mM KCl, 5 mM DTT, and 0.01% Triton X-100. Furthermore, the RNMT activity was not affected by up to 10% DMSO under the optimized assay conditions (Fig. 1I).

Overall, the assay optimization led to a 50% increase in assay signal over the starting assay conditions (Suppl. Fig. S2A).

### Kinetic characterization of RNMT

**Figure 2:**
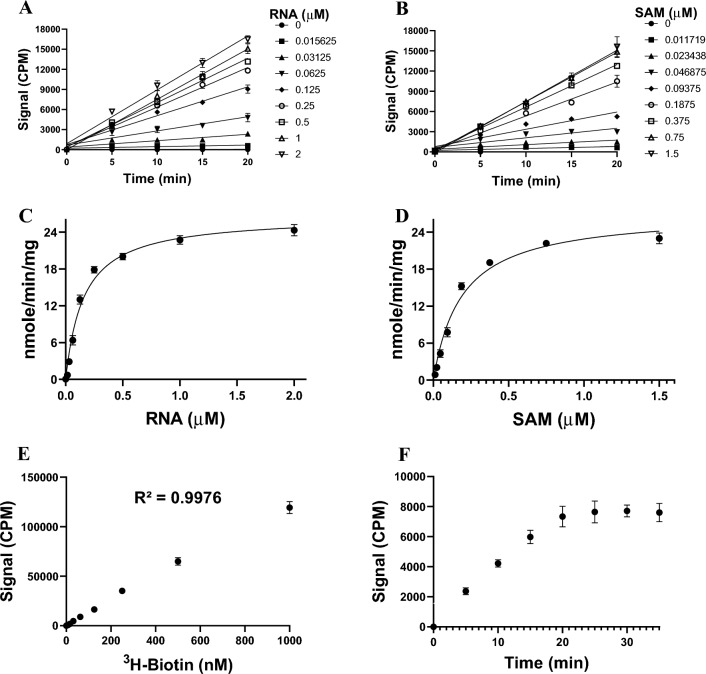
Kinetic parameter determination for RNMT. The initial velocities were determined at 5 nM RNMT using (**A**) various concentrations of RNA (0.016–2 µM) and a fixed ^3^H-SAM concentration (1.5 µM), and (**B**) varying concentrations of ^3^H-SAM (0.012–1.5 µM) and a fixed RNA concentration (1 µM) under the optimized assay conditions. Linear initial velocities for the first 20 min from A and B were used to calculate the *K*_*m*_ values for (**C**) RNA substrate, and (**D**) SAM. A standard ^3^H-biotin titration curve was generated and used for calculating the *k*_cat_ values. (**F**) The linearity of RNMT reaction was maintained for about 20 min using the optimized reaction conditions. Plotted values are the mean ± standard deviation of three independent experiments. Data were analyzed using GraphPad Prism software 9.

Initial experiments for MTase activity with variable RNMT (1–476 aa) concentrations, under optimized assay conditions, indicated reaction linearity with up to 5 nM of RNMT (Suppl. Fig. S2B). Using 5 nM of RNMT under optimized assay conditions, the linear initial velocities were calculated and used to determine the kinetic parameters for RNMT. Linearity of initial velocities was measured at variable concentrations of RNA and fixed ^3^H-SAM concentration (1.5 µM) (Fig. 2A), and at variable concentrations of ^3^H-SAM and a fixed concentration of RNA (1 µM) (Fig. 2B). Using slopes of the linear initial velocities, apparent *K*_*m*_ (*K*_*m*_^app^) values of 158 ± 8 nM (*k*_*cat*_^*app*^ of 91 ± 1.2 h^-1^) and 190 ± 38 nM (*k*_*cat*_^*app*^ of 93 ± 8 h^-1^) were determined for RNA and ^3^H-SAM, respectively (Fig. 2). We also investigated the activity of the catalytic domain of RNMT (123–476 aa; Suppl. Fig. S3). The linear first 20 min of the reactions (Suppl. Fig. S3A and S3B) was used to calculate the kinetic parameters for the catalytic domain (Table 1). The *K*_*m*_^*app*^ values of 75 ± 11 nM (*k*_*cat*_^*app*^ of 39 ± 2 h^-1^) and 196 ± 10 nM (*k*_*cat*_^*app*^ of 41 ± 2 h^-1^) were determined for RNA and ^3^H-SAM, respectively (Suppl. Fig. S3).

**Table 1:** Kinetic parameters of RNMT. All experiments were performed in triplicate, and data are shown as the mean ± standard deviation.

RNMT	Substrate	*K*_*m*_^*app*^ (nM)	*k*_*cat*_^*app*^ (h^-1^)	*k*_*cat*_/*K*_*m*_ (h^-1^ nM^-1^)
RNMT (1–476)	^3^H-SAM	190 ± 38	93 ± 8	0.48
	RNA	158 ± 8	91 ± 1.2	0.58
RNMT (123–476)	^3^H-SAM	196 ± 10	41 ± 2	0.21
	RNA	75 ± 11	39 ± 2	0.52

### Screening amenability of the RNMT assay

**Figure 3:**
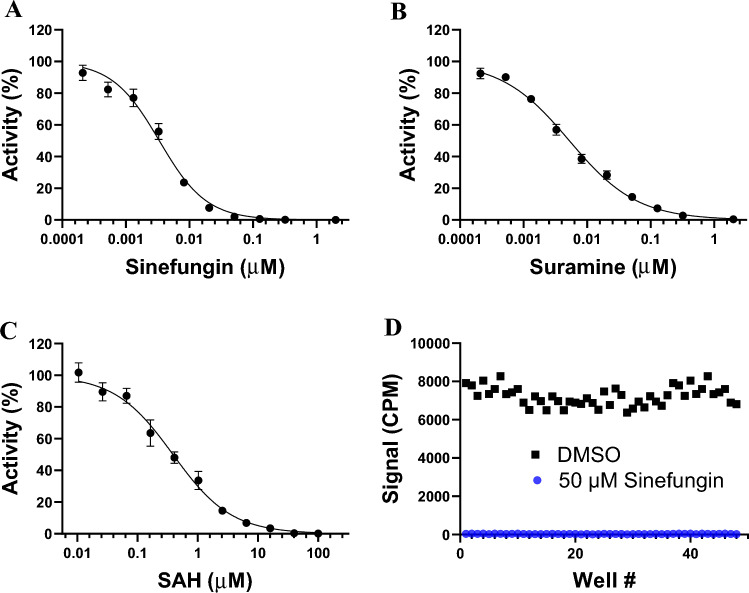
Amenability of the optimized assay for screening. (**A**) Sinefungin, (**B**) Suramin, and (**C**) SAH inhibited RNMT with IC_50_ values of 3 ± 0.6 nM (Hill Slope: 1.2), 5 ± 0.4 nM (Hill Slope: 0.8) and 389 ± 110 nM (Hill Slope: 0.9), respectively. Plotted values are the mean ± standard deviation of three independent experiments. Data were analyzed using GraphPad Prism software 9. (**D**) The Z-factor was determined in the absence (black rectangles) and presence (blue circles) of 50 μM Sinefungin using the optimized screening conditions.

Screening for small molecules is typically performed at *K*_m_ of the substrates to allow potential inhibitors to compete with the substrates for binding to the protein target. The enzyme activity should be linear during such assay period. Testing the RNMT (1–476 aa) activity at 150 nM RNA and 200 nM ^3^H-SAM indicated that the reaction can be run for at least 20 min while maintaining linearity (Fig. 2F). To check the quality of the optimized assay, effect of the pan inhibitors of methyltransferases such as sinefungin, suramin and SAH (the product of the reaction) on RNMT activity was assessed, and IC_50_ values of 3 ± 0.6 nM (Hill Slope: − 1.2), 5 ± 0.4 nM (Hill Slope: − 0.8) and 389 ± 110 nM (Hill Slope: − 0.9) were determined, respectively (Fig. 3A–C). Robustness of the optimized assay for screening in 384 well format was also confirmed by Z-factor determination (0.79, Fig. 3D). Z-factor is a dimensionless screening window coefficient that is meaningful within the range of − 1 < and ≤ 1, and reflective of both the assay dynamic range and the data variation associated with the signal measurement. Typically, the assays with Z-factors of higher than 0.5 are considered reliable for screening. However, the higher the Z-factors, the better and more reproducible the assay is for high-throughput screening (larger dynamic range and/or smaller data variability)^25^.

The RNA m7G cap formation by RNMT plays critical roles in RNA processing, modification, translation, and protecting RNA from degradation in mammalian cells^2,5^. The availability of RNMT assays suitable for screening small molecule inhibitors is critical for development of potent, selective and cell active small molecules (chemical probes) to further investigate RNMT roles in cancers^13,14, 26^. The viral capping enzymes have also been attractive targets for drug discovery aimed at blocking cap formation during replication of human infecting viruses^8,24^. Determining the selectivity of increasing number of SARS-CoV-2 nsp14 inhibitors against human RNMT would be essential in development of antiviral therapeutics^23,27–29^. In this study, we report development of a radiometric assay for RNMT suitable for screening small molecule libraries to identify modulators of its activity, and to employ for selectivity determination of inhibitors of SARS-CoV-2 methyltransferases (nsp14 and nsp16)^29^.

It was previously reported that catalytic domain of RNMT is mainly responsible for cap methyltransferase activity while its N-terminal domain is required for RNMT recruitment to transcription initiation sites and is not essential for methyltransferase activity^30^. Determination of the kinetic parameters for both full-length and catalytic domain of human RNMT (Table 1) show that catalytic domain is highly active with some differences in apparent kinetic parameters compared to the full-length. This may affect decision making on which construct to use for screening or determination of IC_50_ values for small molecule inhibitors. The k_cat_^app^ values for the full length RNMT was almost twofold higher than catalytic domain. Although the K_m_^app^ values for SAM were similar for both constructs, and K_m_^app^ for the RNA was about twofold lower with the catalytic domain (Table 1). As we may prefer to use the full-length construct for selectivity assays and IC_50_ determination or even screening, which will be closer to in cell conditions, one may also use the catalytic domain purified from E. coli expression by adjusting the concentration of both SAM and RNA accordingly. In this case, it would be important to test all hits against the full-length protein as well along with performing orthogonal confirmation and selectivity assays.

The inhibitory effect of the pan-MTase inhibitors (sinefungin, suramin and SAH) against RNMT was confirmed using this assay. Sinefungin inhibited RNMT activity with IC_50_ value of 3 ± 0.6 nM which is reasonably close to the previously reported value for inhibition of yeast cap MTase Abd1 by sinefungin (IC_50_ of 24 nM)^18^. Suramin and SAH also inhibited RNMT potently with IC_50_ values of 5 ± 0.4 nM (Hill Slope: − 0.8) and 389 ± 110 nM (Hill Slope: − 0.9), respectively. These data along with Z-Factor of 0.79 for screening in 384-well format further supports suitability of this optimized assay for medium and high-throughput screening.

## Conclusion

We have developed and optimized a radiometric assay for human RNMT, determined its kinetic parameters, and optimized the assay for high-throughput screening. The assay will enable identifying and characterizing potent inhibitors towards development of chemical tools to investigate the roles RNMT may play in various cancers, and development of selective antiviral therapeutics. Identified SARS-CoV-2 methyltransferase inhibitors could be tested to increase selectivity against human RNMT, reducing potential side effects of nsp14/nsp16 inhibition-based therapeutics.

## Materials and methods

### Reagents

S-adenosyl-L-methionine (^3^H-SAM), and the 384 and 96 well Streptavidin PLUS High-Capacity FlashPlates, (cat. #: SMP410A001PK) were purchased from PerkinElmer (Massachusetts, USA). Biotin-labeled single-strand RNA (5′ GpppACCCCCCCCC-Biotin 3′), referred to as RNA substrate here on, was custom synthesized by TriLink BioTechnologies (San Diego, USA). All RNA solutions were prepared by solubilizing in nuclease-free water in the presence of RNAseOUT™ recombinant ribonuclease inhibitor (Thermo Fisher, Cat. #. 10,777,019) at a final concentration of 0.4 U/µL. S-adenosylhomocysteine (SAH), suramin and sinefungin were purchased from Sigma-Aldrich (Missouri, USA).

### Protein expression and purification

DNA fragments encoding the RNMT residues 1–476 amplified by PCR and sub-cloned into pFBOH-MHL downstream of the HisTag. The resulting plasmid was transformed into DH10Bac™ Competent *Escherichia coli* (Invitrogen), a recombinant viral DNA bacmid was purified, followed by a recombinant baculovirus generation in sf9 insect cells. sf9 cells grown in HyQ® SFX insect serum-free medium (Thermo Scientific) were infected with 10 ml of P3 viral stock per 0.8 L of suspension cell culture and incubated at 27 °C using a platform shaker set at 100 RPM. The cells were collected after 72 h of post infection time, when viability dropped to 70–80%.

Cells were harvested and re-suspended in 50 mM Tris–HCl buffer, pH 8, containing 500 mM NaCl, 5 mM imidazole and 5% glycerol, 1X protease inhibitor cocktail (100 X protease inhibitor stock in 70% ethanol (0.25 mg/ml Aprotinin, 0.25 mg/ml Leupeptin, 0.25 mg/ml Pepstatin A and 0.25 mg/ml E-64) or Roche complete EDTA-free protease inhibitor cocktail tablet. The cells were lysed chemically by rotating 30 min with NP40 (final concentration of 0.6%), 120 µL Benzonase nuclease (in house) and 1 mM TCEP followed by sonication at frequency of 7.5 (10″ on/10″ off) for 5 min (Sonicator 3000, Misoni). The crude extract was clarified by high-speed centrifugation (60 min at 36,000 × g at 4 °C) by Beckman Coulter centrifuge.

The clarified lysate was then passed through a pre-equilibrated Ni–NTA resin (Qiagen) column. The column was washed and eluted by running 50 mM Tris–HCl (pH 8.0), 500 mM NaCl, 5% glycerol, containing 30 mM and 250 mM imidazole, respectively. The eluent was further purified by gel filtration on a Superdex200 26/60 using an ÄKTA purifier (GE Healthcare) pre equilibrated with 50 mM Tris pH 8, 150 mM NaCl, 5% glycerol, 1 mM TCEP. The molecular weight of RNMT was confirmed by running 10 μg of protein on mass spectrometer (Agilent Technologies, 6545 Q-TOF LC/MS). The purity of the fractions was confirmed on SDS-PAGE gels and the pure fractions were pooled, concentrated and flash frozen (Fig. S1).

DNA fragment encoding the catalytic domain of RNMT (123–476 aa) was amplified by PCR and sub-cloned into pET28a-LIC (https://www.thesgc.org/sites/default/files/toronto_vectors/pET28a-LIC.pdf) downstream of the HisTag. Following transformation into *E. coli* BL21 (DE3), RNMT (123–476 aa) was over-expressed at 37 °C by inoculating Terrific Broth with overnight culture, both supplemented with 50 µg/ml Kanamycin and 35 µg/ml chloramphenicol. When the OD600 of the culture reached 0.8–1.5, the temperature was lowered to 18 °C, the culture was induced with 0.5 mM IPTG (isopropyl-1-thio-D-galactopyranoside) and incubated overnight before being harvested (7000 rpm for 10 min at 4 °C) using a Beckman Coulter centrifuge. Harvested cells were re-suspended in 20 mM Tris–HCl, pH 7.5, containing 500 mM NaCl, 5 mM imidazole and 5% glycerol, 1× protease inhibitor cocktail (100 × protease inhibitor stock in 70% ethanol (0.25 mg/ml Aprotinin, 0.25 mg/ml Leupeptin, 0.25 mg/ml Pepstatin A and 0.25 mg/ml E-64) or Roche complete EDTA-free protease inhibitor cocktail tablet. The cells were lysed chemically by rotating 30 min with CHAPS (final concentration of 0.5%) and 5 µl/L Benzonase Nuclease (in house) followed by sonication at frequency of 8 (10″ on/10″ off) for 4 min (Sonicator 3000, Misoni). The crude extract was clarified by high-speed centrifugation (60 min at 36,000 × g at 4 °C) by Beckman Coulter centrifuge. The clarified lysate was then passed through a pre-equilibrated Ni–NTA resin (Qiagen) column. The column was washed and eluted by running 50 mM Tris–HCl pH 8, 500 mM NaCl, 5% glycerol, containing 30 mM and 250 mM imidazole, respectively. The eluent was further purified by gel filtration on a Superdex200 16/60 column using an ÄKTA purifier (GE Healthcare) pre equilibrated with 50 mM Tris pH 8, 500 mM NaCl, 1 mM DTT. The purity of the fractions was confirmed on SDS-PAGE gel (Fig. S1) and the pure fractions were pooled, concentrated and flash frozen for further use.

### Optimization of assay conditions for RNMT

The methyltransferase activity of RNMT was measured using a radiometric assay. The transfer of ^3^H -methyl group from ^3^H-SAM to the RNA substrate (5′ GpppACCCCCCCCC-Biotin 3′) was monitored using a scintillation proximity assay (SPA). Unless stated otherwise, all experiments were performed in a total assay volume of 20 µL in 384 or 96 well plates in triplicate at room temperature (23 °C). 20 μL mixtures containing 10 mM Tris (pH 7.5), 250 μM MgCl_2_, 10 mM KCl, 5 mM DTT, 0.01% Triton X-100, 5 nM of RNMT and 150 nM RNA substrate were prepared. The reactions were started by the addition of 250 nM ^3^H-SAM. Reactions proceeded for 30 min and then quenched by adding 10 μL of 7.5 M Guanidine hydrochloride followed by 160 μL of 20 mM Tris–HCl (pH 8.0). The reaction products were then transferred into Streptavidin-coated FlashPlates for scintillation counting using a TopCount instrument (PerkinElmer, Massachusetts, USA). To determine the optimum buffer pH, 10 mM Tris–HCl was used to generate the pH profile ranging from 6.5 to 9.0. The effect of various reagents such as salts, detergents, reducing agents, BSA, EDTA, and DMSO was investigated through titration of each reagent in assay buffer containing 5 nM RNMT, 150 nM RNA, and 250 nM ^3^H-SAM at pH 7.5 and measuring their relative activity compared to the control (i.e., reactions without additive) using the SPA based assay. The following buffer was chosen as the optimal reaction condition: 10 mM Tris (pH 7.5), 250 μM MgCl_2_, 10 mM KCl, 5 mM DTT, 0.01% Triton X-100. All subsequent experiments were performed using this buffer condition, at room temperature (23 °C).

### Kinetic characterization of RNMT

Apparent kinetic parameters (*K*_m_^app^ and *k*_cat_^app^) of RNMT were determined using a series of reactions under optimized buffer condition in triplicate in standard 96-well polypropylene plates containing 5 nM RNMT at saturating concentration of one substrate (1 µM RNA or 1.5 µM ^3^H-SAM) and varying concentration of the other (from 12 to 1500 nM for ^3^H-SAM and from 16 to 2000 nM for RNA). Reactions were quenched at various time points (5, 10, 15, 20, 30, 40, and 60 min) by adding 10 µL of 7.5 M Guanidine hydrochloride and 160 µL of 20 mM Tris–HCl (pH 8.0) in each well followed by transfer into 96-well FlashPlates. ^3^H-Biotin at different concentrations was used as a control. After overnight incubation, the level of ^3^H-methylated-RNA was measured by scintillation counting as CPM (counts per minute). Initial velocities of the reaction were calculated from the linear portion of the reaction curves (Fig. 2A,B) and were plotted as a function of each substrate concentration (Fig. 2C,D) (^3^H-SAM and RNA) to determine their *K*_m_^app^ values using Michaelis–Menten equation by GraphPad® Prism 9 Software. Maximum velocity (V_max_) obtained from the Michaelis–Menten plot was used to calculate the apparent turnover number (*k*_cat_^app^). Here are the equations for calculation of *K*_m_^app^ and *k*_cat_^app^:

$${\text{V}} = \frac{{V_{\max }^{{{\text{app}}}} [{\text{S}}]}}{{K_{m}^{app}+ [{\text{S}}]}}$$

where V is the velocity, *V*_max_^app^ is the apparent maximum velocity, and *K*_m_^app^ is the apparent Michaelis–Menten constant under the specified assay conditions used in this experiment. [S] is substrate concentration.

$$k_{{{\text{cat}}}}^{{{\text{app}}}} = V_{{{\text{max}}}}^{{{\text{app}}}} /\left[ {\text{E}} \right],$$

where [E] is the concentration of enzyme.

### IC_50_ determination

SAH, Sinefungin, and Suramin were tested at various concentrations from 5 nM to 50 µM final concentration to determine their half-maximal inhibitory concentration (IC_50_) values. The final DMSO concentration was 2%. The final reaction mixture consisted of 5 nM RNMT, 200 nM ^3^H-SAM, 150 nM RNA in 10 mM Tris–HCl pH 7.5, 250 µM MgCl_2_, 10 mM KCl, 5 mM DTT and 0.01% Triton X-100. Reaction time was 20 min. Data were fitted to Four Parameter Logistic equation using the GraphPad® Prism 9 Software.

### Z-factor determination

Standard Z-factor determination was performed to evaluate the effectiveness of the RNMT assay for screening purposes. The optimized reaction mixture containing 5 nM RNMT, and 150 nM RNA was prepared in the presence and absence of 50 μM sinefungin in 384-well format using an Agilent Bravo automated liquid-handling instrument. The final DMSO concentration was 2%. The reactions were started by the addition of 200 nM ^3^H-SAM and were incubated for 20 min at 23 °C. After measuring the signal by the SPA-based method, the Z-factor was calculated as previously described^25^. Briefly, we used the following equation for calculating the Z-factor:

$$Z = 1 - \frac{{3{\text{SD}}\;{\text{of}}\;{\text{sample}} + 3{\text{SD}}\;{\text{of}}\;{\text{control}}}}{{{\text{mean}}\;{\text{of}}\;{\text{samble}} - {\text{mean}}\;{\text{of}}\;{\text{control}}}}$$

### Supplementary Information

Supplementary Information 1.

Supplementary Figures.

## Data Availability

All supporting data related to this manuscript are available through the Supplementary files.
