# Supplementary material for: Kinetic characterization of human mRNA guanine-N7 methyltransferase
Source: Sci Rep. 2024 Feb 24;14:4509. doi: 10.1038/s41598-024-55184-5 (PMC10894281; doi:10.1038/s41598-024-55184-5)
Supplement: Supplementary file 2 — Supplementary Figures. [file 41598_2024_55184_MOESM2_ESM.pdf]

Supplementary Data

Kinetic characterization of human mRNA guanine-N7 methyltransferase

Sumera Perveen[1], Aliakbar Khalili Yazdi[1], Taraneh Hajian[2], Fengling Li[1], and Masoud Vedadi[2,3,4*]

[1]Structural Genomics Consortium, University of Toronto, Toronto, Ontario, M5G 1L7, Canada

[2]Ontario Institute for Cancer Research, 661 University Ave, Toronto, ON, M5G 0A3, Canada

[3]Department of Pharmacology and Toxicology, University of Toronto, Toronto, Ontario, M5S 1A8, Canada

[4]QBI COVID-19 Research Group (QCRG), San Francisco, California 94158, United States.

*To whom correspondence should be addressed:

Masoud Vedadi; Tel.: 416-432-1980; E-mail: m.vedadi@utoronto.ca

Table of content **Page**

**Figure S1:** Purification of RNMT.	**S2**
**Figure S2.** Assay optimization.	**S3**
**Figure S3:** Kinetic characterization of RNMT (123-476).	**S4**

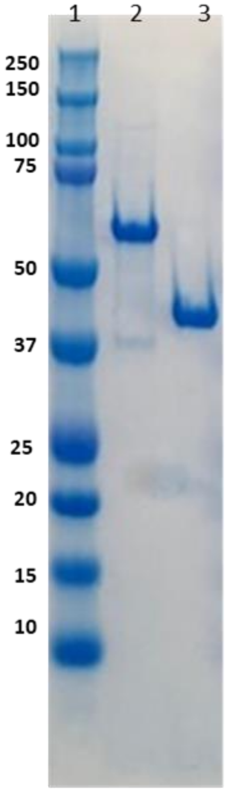

Figure S1. Purification of RNMT. Proteins were run on SDS-PAGE (4-12% Bis-Tris gel; Life Technologies). Lanes are (1) Bio-RAD Precision Plus Protein Standards, (2) Full length RNMT (1-476 AA), and (3) Catalytic domain of RNMT1 (123-476 AA). Unmodified full image of the original gel taken by iPhone camera is presented below. Presented lanes in this figure are 11, 12, and 13. The section was cropped using Microsoft Paint with no picture modification.

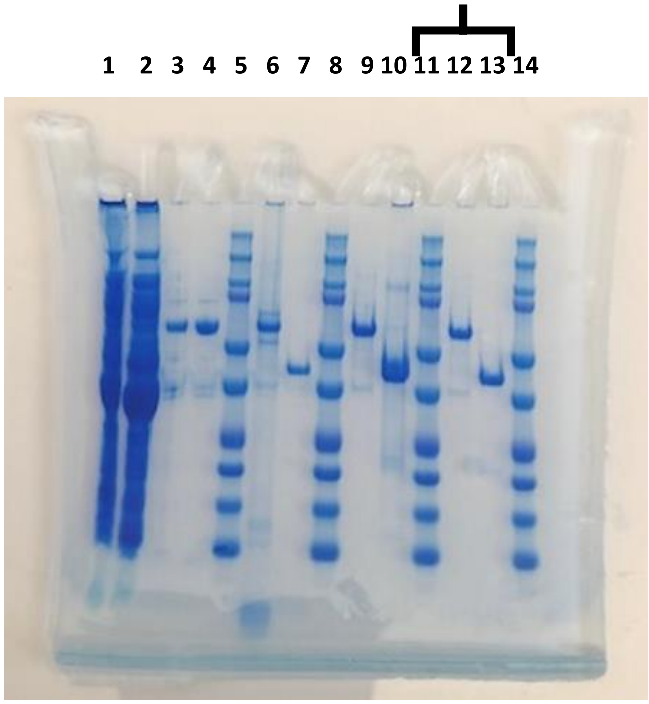

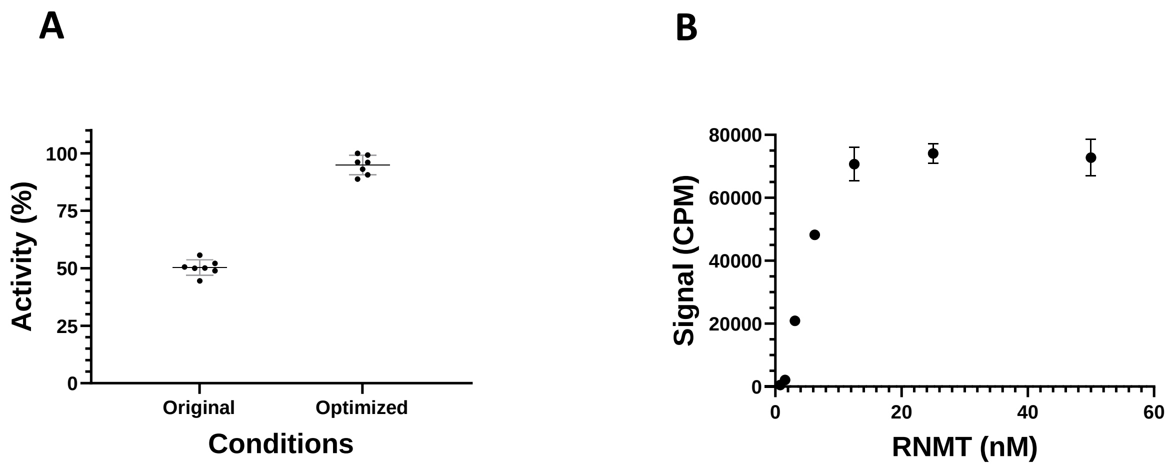

Figure S2. Assay optimization for RNMT. (A) RNMT activity comparison in the original buffer (20 mM Tris HCl, pH 7.0, 5 mM DTT) versus the optimized buffer condition (10 mM Tris HCl pH 7.5, 250 µM $MgCl_2$, 10 mM KCl, 5 mM DTT, and 0.01 % Triton X-100), using 5 nM RNMT, 150 nM RNA and 200 nM ^{3}H-SAM. Data presented is from eight independent experiments (n=8). (B) RNMT activity at various concentrations of RNMT (0.8 nM to 50 nM), using 0.5 µM RNA and 2 µM ^{3}H-SAM. Plotted values are the mean ± standard deviation of three independent experiments. Data were analyzed using GraphPad Prism software 9.

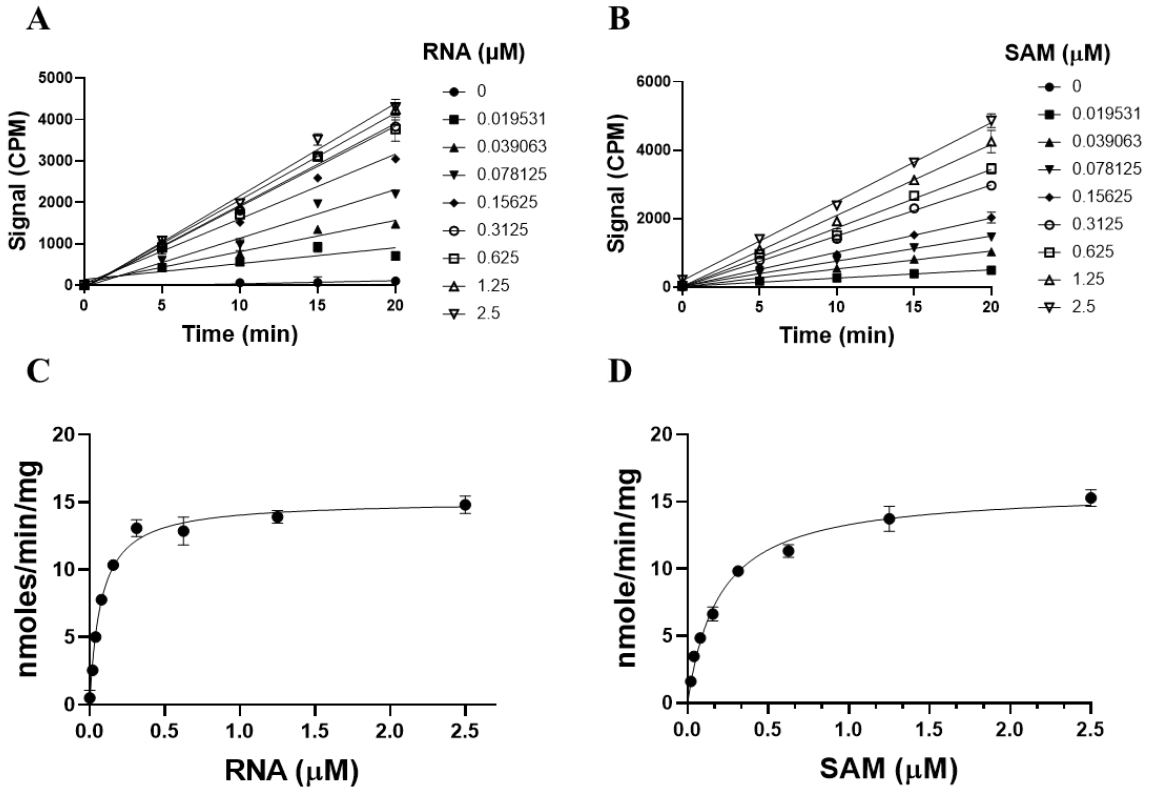

Figure S3. Kinetic parameter determination for catalytic domain of RNMT (123-476). The initial velocities were determined by using 3 nM RNMT (123-476) at (A) various concentrations of RNA (2.5 µM-0.0195 µM) and a fixed ^{3}H-SAM concentration (1.5 µM) and (B) varying concentrations of ^{3}H-SAM (1.5 µM-0.0117 µM) and a fixed RNA concentration of 1 µM under the optimized assay conditions. Linear initial velocities for the first 20 min from A and B were used to calculate the K_m values for (C) RNA substrate and (D) ^{3}H-SAM. Plotted values are the mean ± standard deviation of three independent experiments. Data were analyzed using GraphPad Prism software 9.
